# Revisiting plant hardiness zones to include multiple climatic stress dimensions

**DOI:** 10.1016/j.isci.2024.110824

**Published:** 2024-08-26

**Authors:** Narayani Barve, Uzma Ashraf, Vijay Barve, Marlon E. Cobos, Claudia Nuñez-Penichet, A. Townsend Peterson

**Affiliations:** 1 Florida Museum of Natural History, University of Florida, Gainesville, FL, USA; 2 Department of Ecology and Evolutionary Biology, University of Tennessee, Knoxville, TN, USA; 3 Department of Land, Air and Water Resources, University of California, Davis, Davis, CA, USA; 4 Wild Energy Center, Energy and Efficiency Institute, University of California, Davis, Davis, CA, USA; 5 Marine Biodiversity Center, Natural History Museum of Los Angeles Country, Los Angeles, CA, USA; 6 Department of Ecology and Evolutionary Biology & Biodiversity Institute, University of Kansas, Lawrence, KS, USA

**Keywords:** Ecology, Plant Biology, Botany, Plant ecology, Plant biogeography, Agricultural science, Horticulture

## Abstract

A tradition exists for delineating “hardiness zones” for important plants in horticulture and agriculture. However, these zones are typically based on surviving cold winter conditions, disregarding other stressors. Factors such as the effects of summer heat, aridity, or excessive humidity have been overlooked, limiting our understanding of challenges faced by plants associated with human activities, particularly in a time of rapid global-scale climate change. Annual plants not exposed to winter cold and heat-sensitive plants encountering early summer heat waves may experience significant difficulties. Here, we establish hardiness zone criteria for four climatic dimensions: heat, cold, dryness, and moisture. We explore how this expanded concept of hardiness zones could be implemented in the context of 872 tree species in the United States, as a step toward understanding stressors that plants experience in different climates. The aim is to provide insights that may be informative for horticultural and agricultural practices.

## Highlights

• Traditional hardiness zones are based on the winter cold stress
• Consider heat, cold, dryness, and moisture for plant hardiness zones
• Identifies hardiness zones for 872 tree species in the US
• Provide insights for horticultural and agricultural practices

## Graphical abstract

**Figure:**
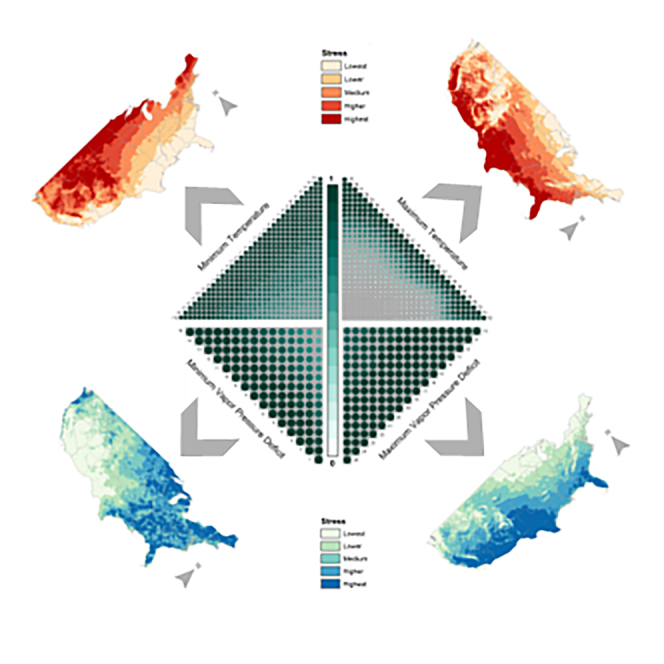

## Introduction

The concept of delimiting zones of differing levels of climate stress for plants has a history dating back almost a century. For example, Rehder^1^ published a map of the United States (US), showing zones appropriate for different sorts of plants, and this map underwent numerous subsequent updates and improvements by personnel at the Arnold Arboretum. Beginning in 1960, the US Department of Agriculture (USDA) adopted the hardiness zone concept; by the 1990s, the USDA had improved the hardiness maps greatly via the incorporation of data from large numbers of weather stations.^2^ The hardiness zone concept is now firmly established in horticulture and vegetation management,^3^ to the point that most seed packets sold commercially have a map of hardiness zones and a rating for that particular plant strain or species regarding in which zones it can thrive.^2^ Throughout this century, the hardiness zone concept has focused almost exclusively on winter cold stress, yet that type of stress is not universally relevant to all plant species.^4^

The fields of distributional ecology and ecophysiology have approached the same basic ideas—i.e., of distributional limitations of species—from a much broader perspective.^5^ In that framework, since the very first papers on the subject,^6^ the multifactorial nature of distributional limitation has been clear, such that many factors apart from winter cold stress are considered.^7^ The expectation is that a single factor may be limiting at any time, but that a distinct factor would lie behind the first and manifest as a constraint when the first limiting factor is removed or overcome. Under this view, multiple interacting factors, such as temperature, moisture, habitat availability, and biotic interactions, collectively shape and limit species’ ranges.^8^ Recognizing the interplay among these factors allows for a more comprehensive assessment of factors that influence a species’ geographic distribution.^9^ Such a broader, holistic perspective not only enhances theoretical understanding but also has practical implications for conservation and management efforts, as it provides a more accurate assessment of the potential impacts of environmental changes on species’ distributions.

The idea that multiple environmental dimensions constrain species’ geographic distributions is not new. One previous study proposed the utility of environmental envelopes (i.e., correlative ecological niche models) to create species-specific hardiness zones.^10^^,^^11^ While we appreciate the creativity of such an approach, we foresee problems with its implementation, since distributional data will often not be available in planted systems, among other complications. Hence, in this study, we have chosen to remain within the general paradigm of hardiness zone maps, but create maps in diverse environmental dimensions. This body of work follows interesting and creative explorations developed in great detail for environments across California.^12^

In this contribution, we expand the traditional notion of hardiness zones by considering multiple environmental dimensions that impact plant survival and growth. We go beyond the conventional focus on winter cold stress to encompass additional factors such as heat, aridity, and humidity. As plants face various challenges throughout their life cycles, limiting analysis to a single stressor overlooks critical factors that influence species’ geographic distributions. We aim to create comprehensive and informative maps that capture the multidimensional nature of environmental stress and its impact on plant distributions.

## Results

### Delimiting stress areas and hardiness zones

**Figure 1:**
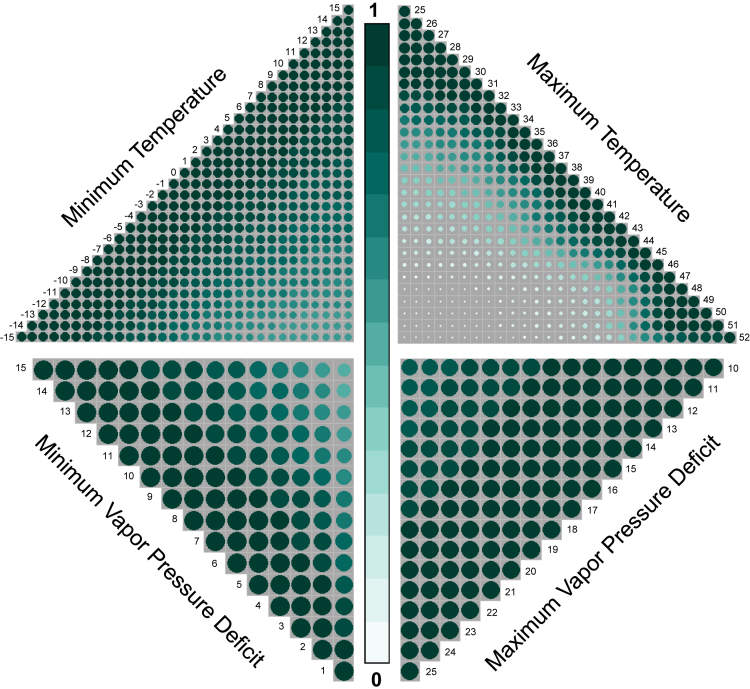
Correlation pattern of climatic variables used in stress class analysis A visual summary of correlation patterns across four thresholds: the top-left quadrant shows correlations involving minimum temperature, the top-right quadrant shows correlations involving maximum temperature, the bottom-left quadrant shows correlations involving minimum vapor pressure deficit, and the bottom-right quadrant shows correlations involving maximum vapor pressure deficit (the bigger and darker circle shows high correlation and vice versa).

Correlation patterns for four environmental variables for various thresholds suggested that correlations are stronger for closer thresholds than for more disparate thresholds (Figure 1), which is expected. This exploration also indicated that no breaks or major discontinuities exist: rather, each threshold represents a point along a gradual spectrum of possible results. Given the result in which thresholds represent points along a continuum, the categorized climatic dimensions of the first component of each of the principal component analyses offer a reasonable suite of stress zones in that climatic dimension. We compared the outcomes of the chi-square analysis with USDA hardiness zones (Figure S1).

**Figure 2:**
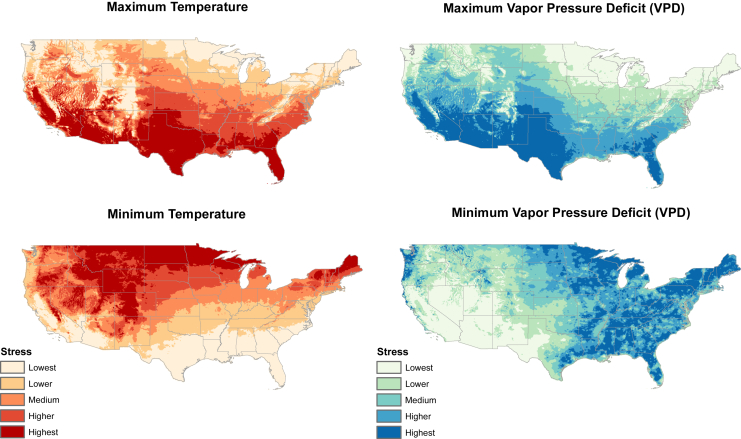
Climatic stress zones in the United States for four environmental dimensions Climatic stress zones are based on quantiles of the first component of principal component analysis (PCA) in each climatic dimension. Stress levels range from lowest to highest, indicated by a gradient from light to dark colors. The geographic variability highlights regions under different degrees of climatic stress. Gray lines represent US state borders.

**Figure 3:**
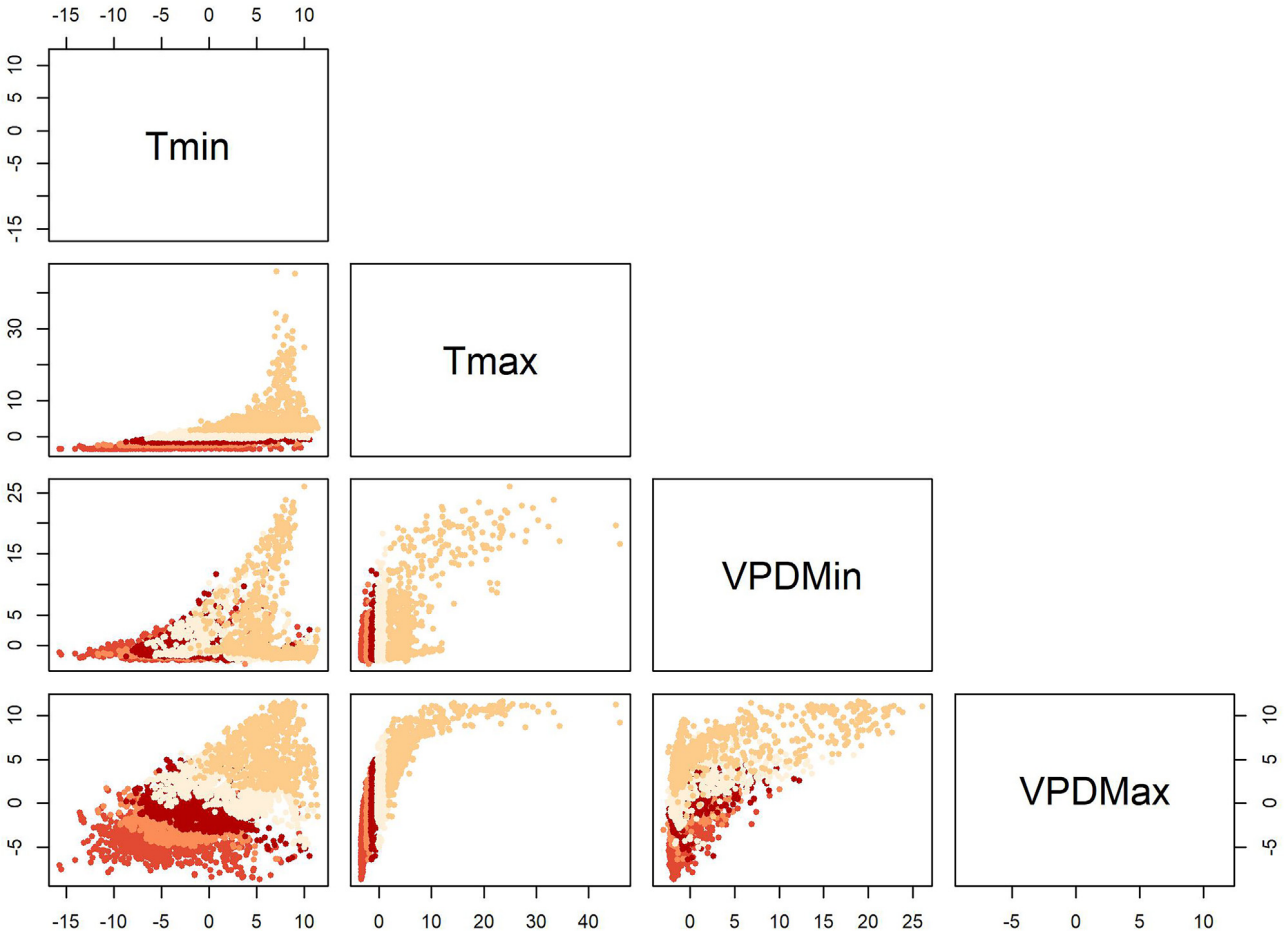
Scatterplot of the first principal component from PCA of climatic variables Scatterplot of the first principal component, derived from PCA of four climatic variables: minimum temperature (Tmin), maximum temperature (Tmax), minimum vapor pressure deficit (VPDmin), and maximum vapor pressure deficit (VPDmax). The combined influence of these variables helps to identify patterns and correlations in the climatic data.

The most stressful zone for maximum temperature was in the southwestern US, whereas lowest stress for maximum temperature was in the northern US and the Rocky Mountains. The opposite pattern was manifested for minimum temperature, with greatest stress in northern regions and high mountains and least stress in the southern regions. Stress areas for minimum and maximum vapor pressure deficit (VPD) showed more complex patterns, that is, for minimum VPD (i.e., too much water available), most stressful areas were in the eastern US, and the lowest stress in the western US. For maximum VPD, maximum stress was in desert regions, and Florida, Texas, Nevada, and California. Correlations among these four stress dimensions were marked in the two temperature axes: the highest stress zone for maximum temperature was toward the southwestern US, whereas the converse was the case for minimum temperature (Figure 2). This mirrored relationship did not hold between maximum and minimum VPD stress zones, however, which were markedly different: maximum VPD stress was highest in the southwestern US and Florida, whereas, for minimum VPD, highest stress was across the eastern US (scatterplots for these variables are presented in Figure 3).

### Applying these hardiness zones

We explored the application of our four-dimensional climate stress and hardiness zones to 872 tree species across the lower 48 US. Among the 872 tree species, 62% had distributions concentrated in the lowest stress category for minimum temperature. However, ∼50% of species were concentrated in areas with higher stress levels for maximum temperature; only ∼25% were concentrated in areas with the lowest stress level. Most species were concentrated under the highest stress level for VPD (Figure S2 and Table S1). These rather counter-intuitive associations likely reflect larger-scale biodiversity patterns such as the latitudinal biodiversity gradient, such that more species have relatively southern distributional patterns within our study area.

**Figure 4:**
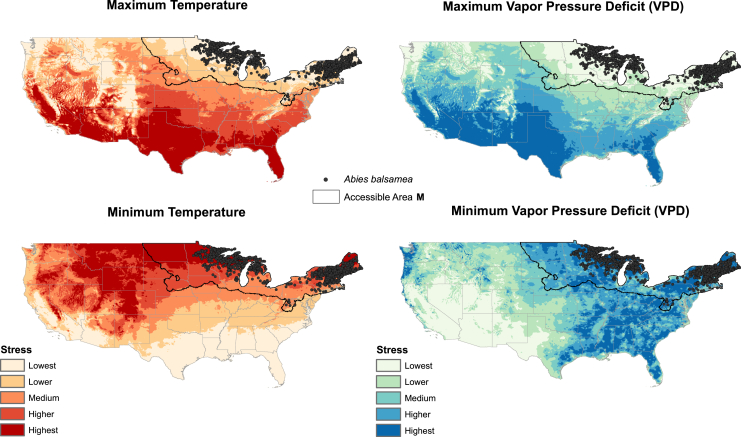
Geographic distribution of *Abies balsamea* concerning climatic stress dimensions Summary of the geographic distribution of *Abies balsamea* concerning four dimensions of climatic stress. In each case, the higher-stress categories are shown in darker shades. As such, this species is associated with stress regarding maximum temperature and maximum vapor pressure deficit (similar illustrations for three other example species are in supplementary materials Figures S3–S5). Area accessible by dispersal, according to Barve et al.^13^ is marked as Accessible Area M with blackline. Gray lines represent US state borders.

For our example species, we explored distributions with respect to the four dimensions of stress and hardiness zones. *Abies balsamea* was concentrated in regions showing the lowest maximum temperature stress and maximum VPD (Figure 4), whereas *Carnegiea gigantea* was concentrated under the lowest stress of minimum temperature stress and minimum VPD (Figure S3). *Juglans nigra* was associated with the lowest stress regarding maximum VPD (Figure S4), whereas *Magnolia grandiflora* was associated with lowest stress in terms of minimum temperature (Figure S5).

## Discussion

### Generalities

Plant hardiness zones are based on the understanding that different plants have different sets of temperature and water-availability tolerances and requirements for their survival, growth, and reproduction.^14^ The utility of hardiness zones in gardening and agriculture lies in their ability to guide plant selection and anticipate the likelihood of a plant’s survival in a particular region. The USDA plant hardiness zones are defined in terms of the average annual minimum temperature over the past 30 years, which indeed is one important factor in a species’ ability to maintain populations across an area.^15^ However, given their focus on temperature, these zones extend in east-to-west bands across the US, despite massive gradients in moisture availability that are often contrary in orientation, running generally northwest to southeast. Consequently, the USDA hardiness zones represent, at best, only a partial view of plant stress and the ability to survive and reproduce^10^ (Indeed, the motivation for this study came from an individual rosemary plant that survived the winter cold only to die in the first summer heat event).

The focus in this contribution has been on plant responses to abiotic dimensions of the environment—i.e., aspects of the environment that are not changed by the presence or absence of the species in question. As such, these “abiotic” dimensions of the environment are more properly termed “non-interactive” environmental dimensions,^5^ and their non-interactive nature makes them far more amenable to being characterized in detail and used in predictive models. A crucial point is that, at least in some cases, the effects of the abiotic/non-interactive variables may be manifested via intermediate, biotically based interactions: e.g., high humidity may affect the populations of a plant species via the effects of some fungus that can grow under those conditions and damage the plants.^5^ Nonetheless, associations between the non-interactive conditions and populations of the species in question, be they direct and proximate or indirect and less proximate, may remain rather constant, such that good predictive ability can be achieved based on the non-interactive dimensions of the environment.

Ray et al. examined global crop yield variability as an effect of variation in climatic conditions. They suggested that crop yield variability is not uniform, but their observations suggested variation in regional crop yields. They also observed that cropwise variability exists in yields.^16^ Agricultural yields can be maintained by adding more water or draining water from soils to mitigate VPD stress levels. However, it is likely to be more difficult to reduce temperature effects on crops as temperature rises due to climate change, as direct manipulation is difficult or impossible.

Although high temperatures may be a variable of particular interest given the difficulty of their control, management steps such as irrigation and soil management may also become more difficult to manage as climate change progresses, which could exacerbate water scarcity and increase temperatures, complicating plant stress management.^17^ Addressing these challenges will strengthen the rationale for the development of a new, more comprehensive hardiness system. In the present contribution, however, we aim only to marshal additional suites of climatic stressors that may be relevant to some plant species.

In horticultural applications, plant injury owing to winter freezing represents a major concern for plant survival.^18^ Indeed, keeping a plant out of stressful conditions concerning other factors (e.g., water and/or humidity requirements) is comparatively easier than avoiding freezing, such as via supplemental watering.^19^ As such, although the USDA hardiness zones are useful, they have significant limitations,^10^ which can be alleviated at least in part by developing an extended hardiness zone concept that incorporates other dimensions of environmental stress. We anticipate that high-heat and low-water-availability conditions will be particularly relevant to many plant species. More generally, a multi-dimensional hardiness zone system can offer a more nuanced understanding of how changing climates may affect plant stress levels through time and across the country.

The application of hardiness zones in agriculture and horticulture can be guided in the broadest-scale sense by the macro-climate dimensions explored in this contribution. Full, predictive implementation of these concepts, however, would involve integrating additional data streams on microclimate, land topology, light intensity, wind speed, vegetation cover, groundwater levels, etc. Our approach is mostly based on thinking about individual plants, rather than agricultural settings characterized by large-scale monocropping practices.^20^ Agricultural fields, unlike natural ecosystems, are cleared and tilled, which alters soil temperature, the seasonality of thaw, and drainage. Agricultural fields are also cleared of competing species (“weeds”) that might be better adapted to prevailing climate conditions than the crops themselves.^21^ Although our methods offer a promising path forward for refining hardiness zones in horticultural contexts, it is crucial to consider differences manifested in agricultural settings when delineating hardiness zones for agricultural applications.

### Steps forward

The next question is how this concept of multidimensional hardiness evaluations could be implemented and made useful to the horticultural and agricultural communities. This system offers important advantages because it marshals considerably more climatic information to plant hardiness classifications, and makes it readily available and tangible to a wide range of potential users. Nonetheless, we anticipate at least two challenges in its broad implementation and use.

The first challenge is the methodological step of establishing which stress/hardiness dimensions are relevant to individual plant species. Only a subset of the four dimensions explored in this study is likely to be relevant to a given species limiting its potential for survival, growth, and reproduction. In this paper, we have presented a simple, correlative approach to stress evaluation for a large number of tree species. Under this approach, counts of known occurrences of each species are compared to null expectations based on area footprints of the hardiness/stress zones to identify which zones hold more or fewer occurrences than would be expected. An alternative approach could be based on linking physiological measurements to stress in larger-scale environmental dimensions, as has been explored for the geographic distribution of Spanish moss (*Tillandsia usneoides*) across the Americas.^22^

The second challenge is how these hardiness/stress zones could be implemented for many species in applied situations. Clearly, as explored earlier, a given plant species will not be likely under stress in all four of the dimensions that we have explored—rather, we expect each plant species to manifest stress in only one or a few dimensions, but not all of them. As such, hardiness zones for individual species would have to be chosen via some algorithm or protocol, and presented flexibly to the user community. For example, lettuce might be limited by maximum temperatures and high VPD (i.e., maximum water stress) but not by the other dimensions. A significant advantage of this multi-factorial classification of hardiness/stress is that users may adjust their plant-care behavior accordingly: one can alleviate stress in maximum VPD via watering, or one can use well-drained soil to avoid stress factors related to minimum VPD.

### Conclusions

This study has explored the potential utility of transferring ideas from the ecological field of distributional ecology to the applied fields of horticulture and agriculture. Both conceptual explorations in distributional ecology and practical experience (e.g., the rosemary plant that survived the winter only to die in the summer) point to a need for an extended hardiness zone concept that goes beyond the limitations of the current USDA hardiness zones. This research path is promising, and future studies should focus on refining these models to address agricultural needs better, ultimately improving crop resilience and agricultural practices.

Future research will need to delve deeper into the specific differences between ecological and agricultural systems and develop models more directly tailored to integrate ecological constraints fully with the peculiarities of agricultural and horticultural systems. Such steps would enable a more comprehensive understanding and practical application of distributional ecology concepts in agriculture. The result would be that of enhancing both theoretical frameworks and practical outcomes for horticulture and agriculture.

### Limitations of the study

This study expands the concept of hardiness zones beyond temperature and precipitation constraints. However, it focuses primarily on ecological applications, which may not directly translate to agricultural contexts. Ecological constraints do not always align completely with agricultural constraints, which present challenges in applying ecological modeling directly to agricultural contexts; examples of these contrasts include frequent supplementation of water or nutrients, or reduction of biotic interactions, in agricultural systems. This contribution does not presume to present a final system; rather, it illustrates what might be developed as a more comprehensive and synthetic version of hardiness zones, and how the zones may be distributed across the lower 48 US states. Our study demonstrates the application of these new hardiness zone concepts to hundreds of tree species, illustrating their potential, but also revealing the complexity and limitations of transferring ecological principles to agriculture. Our observations indicate that many species are not typically found in high-stress zones across all environmental conditions, highlighting the need to develop species-specific hardiness zones.

## Resource availability

### Lead contact

Requests for further information and resources should be directed to the lead contact, Narayani Barve (nbarve@utk.edu).

### Materials availability

This study did not generate new materials.

### Data and code availability

• Data have been downloaded from the PRISM Climate Group and Global Biodiversity Information Facilities, with links provided in the key resources table (KRT).
• Data have been deposited at GitHub and are publicly available. The DOI is listed in the key resources table.
• All original code has been deposited at GitHub and is publicly available. The DOI is listed in the key resources table.
• The output table for all species is provided in Table S1.
• Any additional information required to reanalyze the data reported in this paper is available from the lead contact upon request.

## Acknowledgments

We thank the remaining members of the KUENM working group for their ideas and input in developing this contribution.

## Author contributions

Conceptualization, A.T.P.; investigation, C.N.-P., N.B., and U.A.; formal analysis, V.B., N.B., U.A., and M.E.C.; methodology, A.T.P., N.B., and U.A.; original draft preparation, A.T.P., N.B., and U.A.; writing – review and editing, A.T.P., N.B., U.A., and C.N.-P.; visualization, U.A., N.B., V.B., and C.N.-P.

## Declaration of interests

The authors declare no competing interests.

## STAR★Methods

### Key resources table

**Table:** 

REAGENT or RESOURCE	SOURCE	IDENTIFIER
**Deposited data**		

Climate data	PRISM Climate Group	https://prism.oregonstate.edu/
Occurrence data for 872 tree species	Global Biodiversity Information Facility	https://doi.org/10.15468/dd.fssqjy

**Software and algorithms**		

All analyzed data and code	This study	https://github.com/narayanibarve/Hardiness
R software version 4.3.1	R Core Team	https://www.r-project.org/

### Method details

#### Climatic data and processing

**Figure 5:**
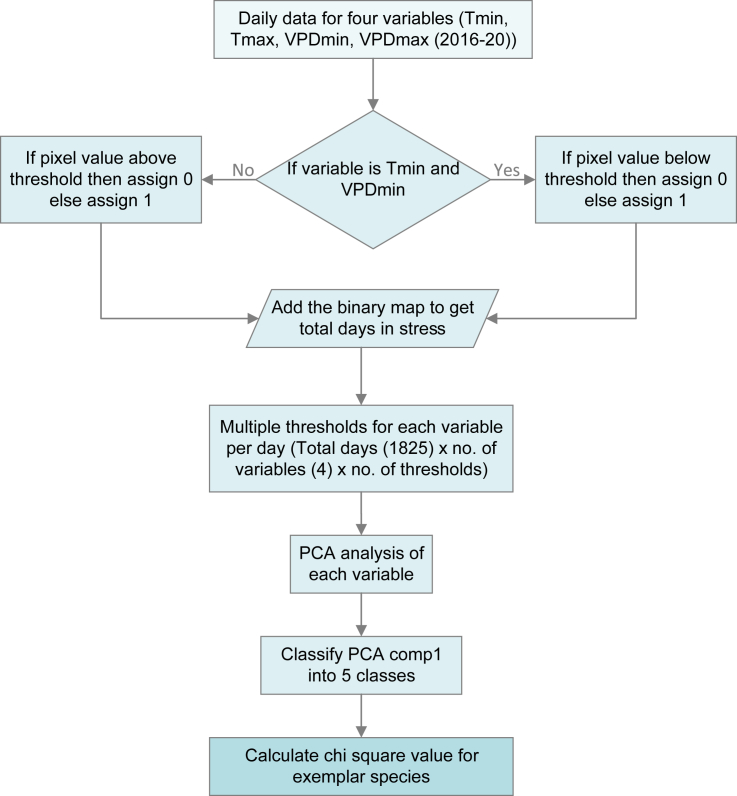
Workflow for generating hardiness zones Flowchart illustrating the step-by-step process for determining the compatibility of species with various stress classes based on each environmental variable (Tmin, minimum temperature; Tmax, maximum temperature; VPDmin, minimum vapor pressure deficit; VPDmax, maximum vapor pressure deficit).

We used the climatic data provided by PRISM Climate Group (https://prism.oregonstate.edu/), given their authoritative, comprehensive, long-term daily data coverage for the Lower 48 US.^23^ We downloaded daily data for minimum temperature, maximum temperature, minimum vapor pressure deficit (VPD), and maximum VPD, for the period from 1 January 2016 to 31 December 2020.^24^ We chose a relatively short time frame (5 years) for averaging daily values to create a “climatology” in view of the nonstationary nature of the current global climate.^25^ A schematic diagram for climatic data processing and selection of hardiness zones is shown in Figure 5.

Instead of using raw values, we focused on threshold values in each climatic dimension and created synthetic indices akin to growing degree days.^26^ As such, we categorized every pixel across the Lower 48 US in a daily data layer for each climatic dimension into suitable (i.e., value 1) *versus* non-suitable (i.e., value 0) pixels based on a threshold value. Because no particular threshold value will be relevant universally to all plant species, we explored all possible values across the ranges -15°C to 15°C for minimum temperature, 25°C to 50°C for maximum temperature, 0-10 for minimum VPD (hPa), and 12-30 for maximum VPD (hPa). For example, for minimum temperature, if the threshold was 5°C, all pixels below 5°C were assigned a value of 0, and all pixels greater than or equal to 5°C were assigned a value of 1. However, for maximum temperature, if the threshold was 12°C, then all pixels with values equal to or below 12°C were assigned a value of 1, and all pixels above 12°C were assigned a value of 0; similar asymmetries were applied to minimum and maximum VPD. These thresholded daily data layers were then summarized in terms of the total number of days (up to 1825) during 2016-2020 meeting each suitability threshold.

We explored these suites of numbers of days meeting thresholds for each of the four climate dimensions. As an initial step, we explored how different thresholds representing stressful conditions were related to one another. Across the study region, as mentioned above, we summed daily climate summaries for different stress thresholds, counting stressful days. We calculated matrices of Pearson correlation coefficients among maps generated for different thresholds for each climate dimension^27^ (Figure 1). Then, we performed a principal components analysis (PCA) separately based on the thresholded maps for each climate dimension to create a threshold-independent summary of the major axes of variation regarding the number of days suitable across different threshold values.^28^ Using the first component of each of these PCAs, we classified the US into five stress levels for each climatic component based on quantiles, such that each class had the same area. We ordered the five classes such that class 1 represented the least stress and class 5 represented the maximum stress in a particular climate dimension.

#### Example applications

Selection of plant hardiness zones for a given species can be done using various methods. For exploration, we analyzed the relative representation of available occurrence data for individual species via chi-square analysis; a better approach would be based on relative performance (e.g., growth rates, reproduction) in different regions, but such data are rarely available across broad regions. We explored two datasets, with and without consideration of species’ accessible area (M, representing the Grinnellian niche concept^13^)—an argument could be developed for either option, as species can occur only within areas accessible to them by their dispersal, yet plant species of interest in horticulture and arboriculture are frequently available for purchase for planting in areas far from their native distributional areas.

The objective is to determine which zones are preferred and which are not based on concentrations of occurrence data in some zones as compared with others. For each climatic dimension, we used chi-square statistics to compare observed and expected numbers of occurrences of the species in each hardiness zone. We calculated the p-values of the chi-square statistics to assess the significance of differences between observed and expected numbers of occurrences in particular zones. Zones showing significant differences between observed and expected distributions with observed exceeding expected numbers are considered preferred zones, whereas zones with no significant difference or significant differences with observed less than expected are deemed not preferred.

To explore this methodology, we used data for all native tree species of the Lower 48 United States, though we concentrated on four species as interesting example cases: *Abies balsamea*, *Carnegiea gigantea*, *Juglans nigra*, and *Magnolia grandiflora*. We used a list of 881 native tree species from Carrero et al. (2022),^29^ and downloaded all occurrence data for those species via the Global Biodiversity Information Facility (GBIF; www.gbif.org; data query DOI is https://doi.org/10.15468/dd.fssqjy). We cleaned the data by removing duplicates, occurrences lacking coordinates, and occurrences with 0,0 coordinates; we also removed apparent outliers (i.e., occurrences falling outside of the area of interest, and occurrences falling far outside the general cloud of occurrences), following Cobos et al. (2018).^30^ We refined occurrence data further by reference to Critchfield and Little (1969).^31^ After the cleaning steps, we had 1,329,120 occurrence data records available for 872 native tree species, which were overlaid with the four synthetic climatic dimensions described above.

The entire dataset (i.e., all 872 native tree species) was explored without consideration of species’ accessible areas (M). The four example tree species were also examined with consideration of preferred and non-preferred hardiness zones based on conditions manifested across their respective accessible areas (Table S1, Figure S2). The M areas for the four species were generated using the records described above, normal environmental variables from the PRISM database^24^ (https://prism.oregonstate.edu/normals/), and the *grinnell* R package.^32^ For *J. nigra* and *M. grandiflora*, records completely outside of their native ranges were excluded. Each set of records was thinned to reduce risks from spatial autocorrelation with a distance of ∼20 km using the *ellipsenm* R package.^33^ We run simulations with combinations of the following parameters: kernel standard deviations (SD) of 3, 4, and 5; 125 and 250 dispersal events; and all other parameters as default. Accessible areas resulting from simulations run with SD = 5 and 250 dispersal events were selected after checking all results.

## Supplemental information

Document S1. Figures S1–S5

Table S1. Compatibility of species with stress zones based on environmental variables, related to Figure 4 Table shows the results for the 872 tree species climate stress zone preferences with four climatic variables: Maximum Temperature (TX), Minimum Temperature (TN), Maximum Vapor Pressure Deficit (VX), Minimum Vapor Pressure Deficit (VN); whereas the five stress classes shows stress gradient from Class 1 (Least Stress) to Class 5 (Maximum Stress).
