# Supplementary material for: Revisiting plant hardiness zones to include multiple climatic stress dimensions
Source: iScience. 2024 Aug 26;27(10):110824. doi: 10.1016/j.isci.2024.110824 (PMC11491728; doi:10.1016/j.isci.2024.110824)
Supplement: Document S1. Figures S1–S5 [file mmc1.pdf]

iScience, Volume *27*

Supplemental information

Revisiting plant hardiness zones to include multiple climatic stress dimensions

Narayani Barve, Uzma Ashraf, Vijay Barve, Marlon E. Cobos, Claudia Nuñez-Penichet, and A. Townsend Peterson

Supplemental Figure

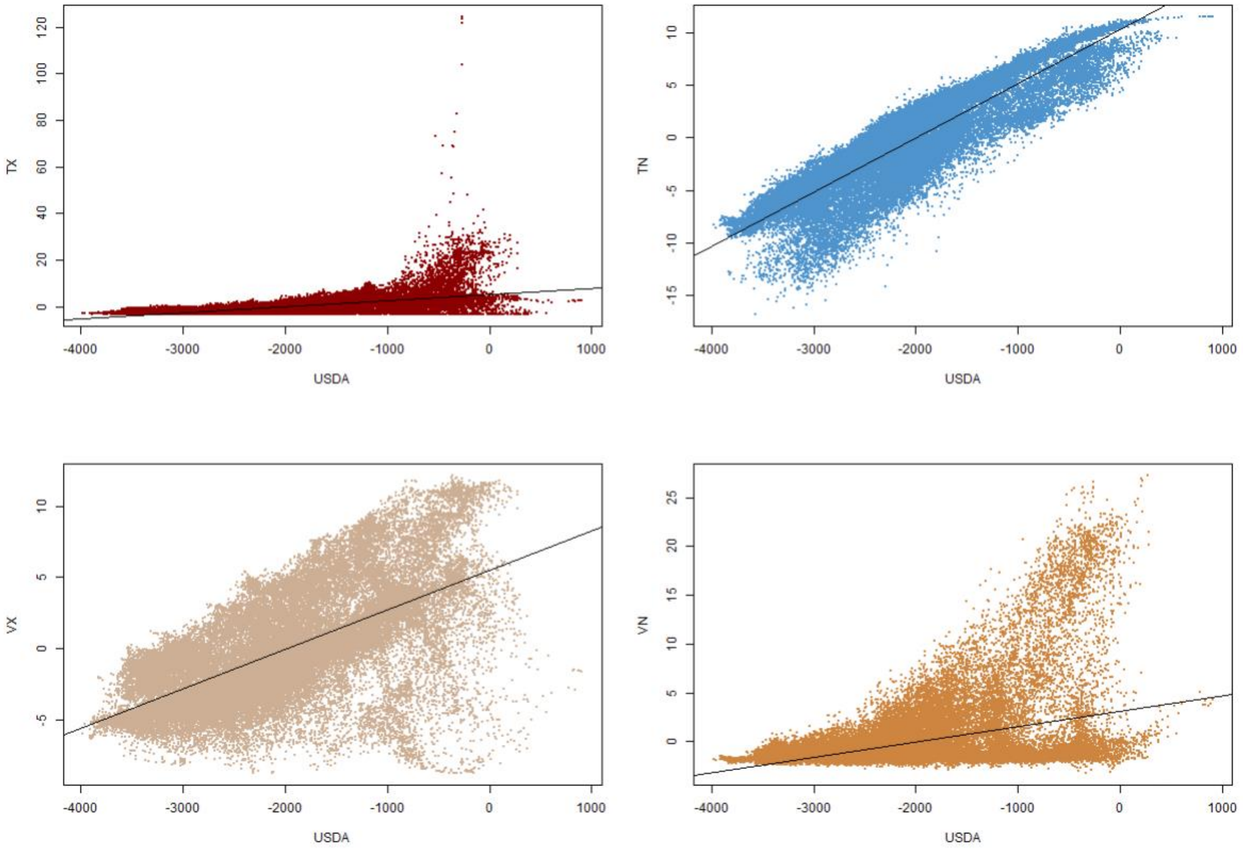

Figure S1 - **Correlation between USDA Hardiness Zones and Climatic Variables, related to Figure 1**

The plots show the relationship between USDA hardiness zones and four climatic variables: maximum temperature (TX), minimum temperature (TN), minimum vapor pressure deficit (VX), and maximum vapor pressure deficit (VN). Each panel illustrates how these variables correlate with USDA hardiness zones, providing insights into the climatic factors influencing plant hardiness.

	Prefers low stress	Prefers high stress	Prefers intermediate stress	No clear preference
Maximum temperature	191	461	163	57
Minimum temperature	523	104	215	30
Maximum vapor pressure deficit	165	413	216	78
Minimum vapor pressure deficit	318	300	188	66

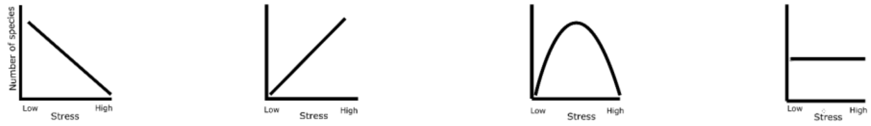

Figure S2 - **Climatic Preferences of 872 Native Tree Species in the Contiguous United States, related to STAR Methods**

The table shows the distribution of tree species based on their preference for different levels of climatic stress. The variables considered are maximum temperature, minimum temperature, maximum vapor pressure deficit, and minimum vapor pressure deficit. Each category (prefers low stress, prefers high stress, prefers intermediate stress, and no clear preference) is illustrated with a corresponding line graph depicting the general trend of species' preferences.

.

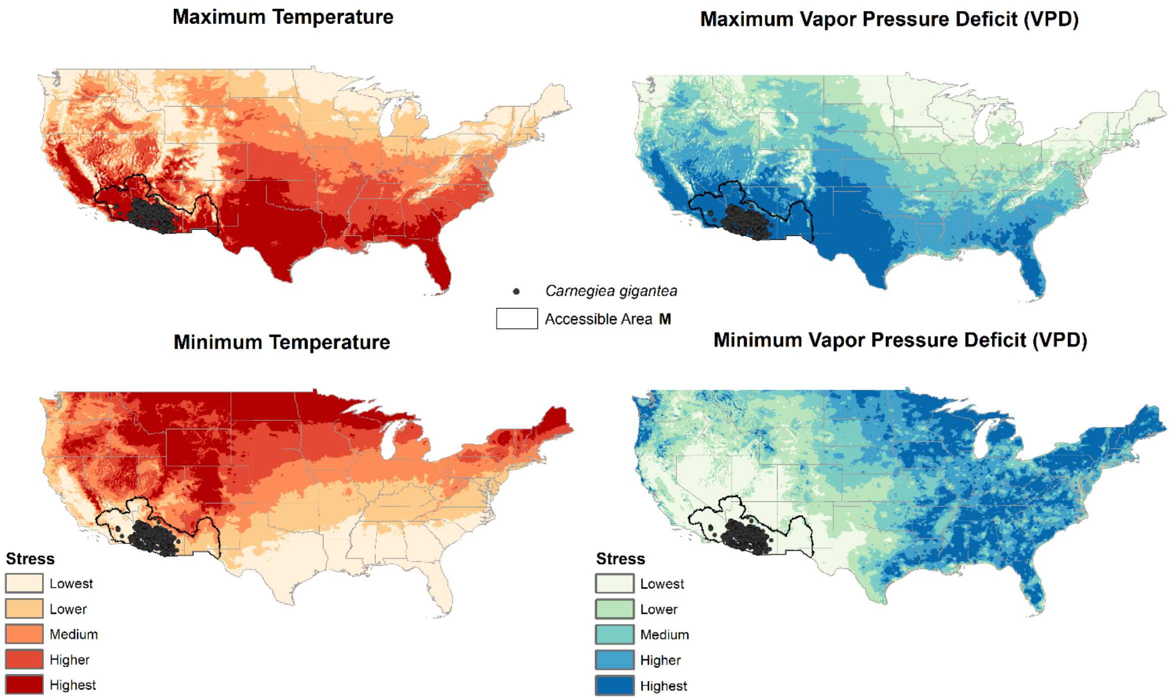

Figure S3 - Distribution of *Carnegiea gigantea* concerning Climatic Stress Dimensions, related to Figure 4

Summary of distribution of *Carnegiea gigantea* with respect to four dimensions of climatic stress. In each case, the higher-stress categories are shown in darker shading. As such, *Carnegiea gigantea* associated with lowest stress zone for minimum temperature and minimum vapor pressure deficit. Accessible area **M** is outlined in black for reference.

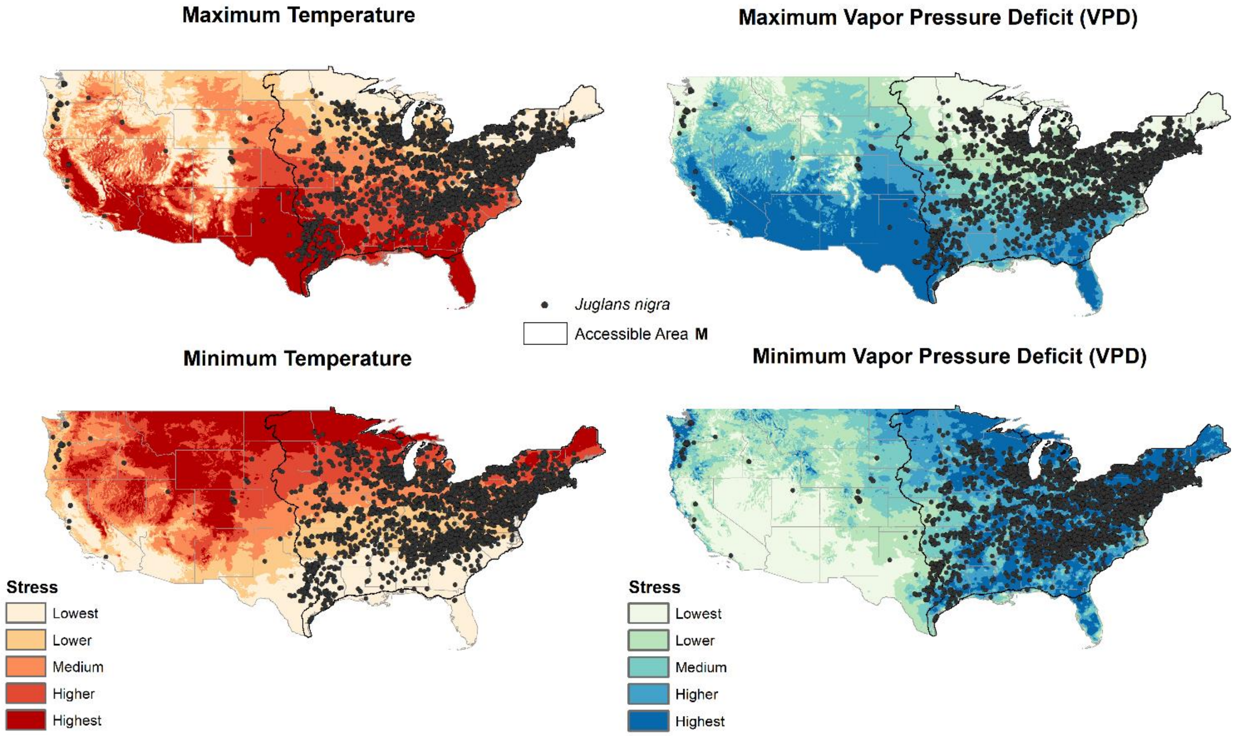

Figure S4 - Distribution of *Juglans nigra* concerning Climatic Stress Dimensions, related to Figure 4

Summary of distribution of *Juglans nigra* with respect to four dimensions of climatic stress. In each case, the higher-stress categories are shown in darker shading. As such, *Juglans nigra* appears to be associated with zones of lowest stress in minimum temperature.

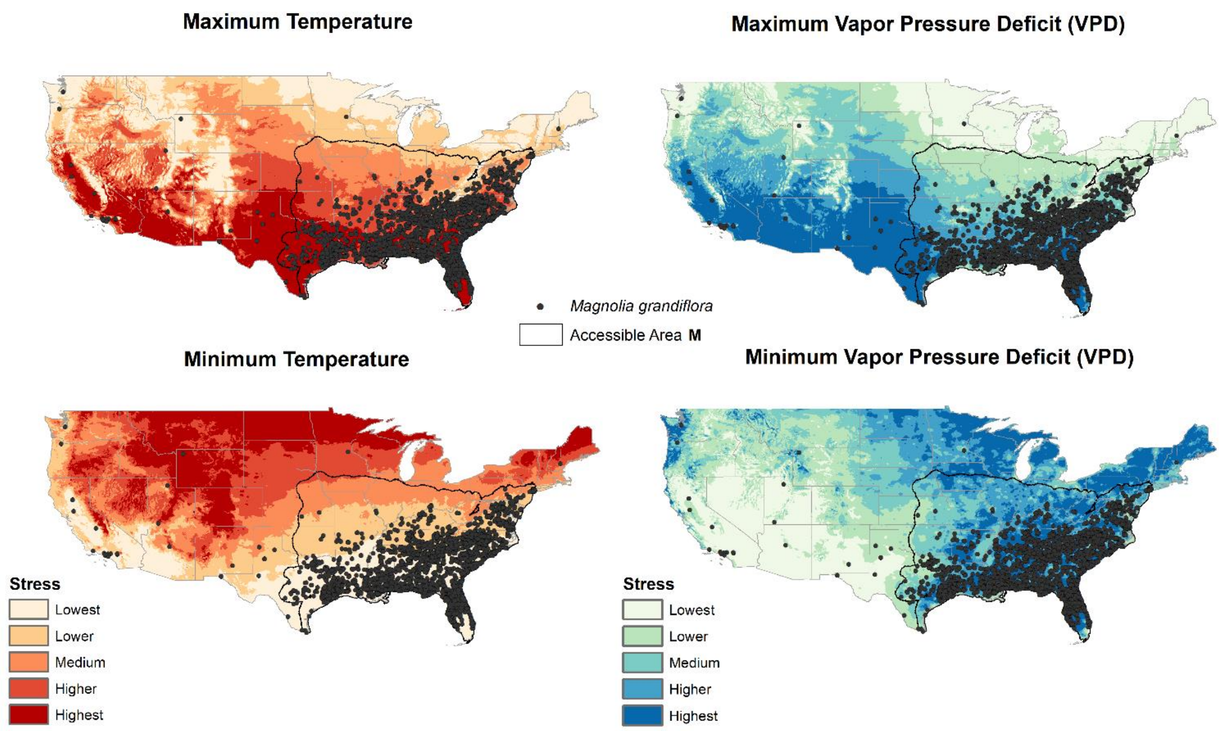

Figure S5 – Distribution of *Magnolia grandiflora* concerning Climatic Stress Dimensions, related to Figure 4

Summary of distribution of *Magnolia grandiflora* with respect to four dimensions of climatic stress. In each case, the higher-stress categories are shown in darker shading. As such, *Magnolia grandiflora* appears to be associated with lowest stress in minimum temperature.
